# Possible Role of Songbirds and Parakeets in Transmission of Influenza A(H7N9) Virus to Humans

**DOI:** 10.3201/eid2003.131271

**Published:** 2014-03

**Authors:** Jeremy C. Jones, Stephanie Sonnberg, Zeynep A. Koçer, Karthik Shanmuganatham, Patrick Seiler, Yuelong Shu, Huachen Zhu, Yi Guan, Malik Peiris, Richard J. Webby, Robert G. Webster

**Affiliations:** St. Jude Children’s Research Hospital, Memphis, Tennessee, USA (J.C. Jones, S. Sonnberg, Z.A. Kocer, K. Shanmuganatham, P. Seiler, R.J. Webby, R.G. Webster);; Chinese Center for Disease Control and Prevention, Beijing, China (Y. Shu);; Shantou University Medical College, Shantou, China (H. Zhu, Y. Guan);; State Key Laboratory of Emerging Infectious Diseases, Shenzhen Third People's Hospital, Shenzhen, China (H. Zhu, Y. Guan, M. Peiris);; The University of Hong Kong, Hong Kong, China (H. Zhu, Y. Guan, M. Peiris)

**Keywords:** influenza virus H7N9, influenza virus avian, influenza virus human, finch, sparrow, budgerigar, songbird, transmission, host intermediate, Passeriformes, Psittaciformes, China, zoonoses, parakeets

## Abstract

Avian-origin influenza A(H7N9) recently emerged in China, causing severe human disease. Several subtype H7N9 isolates contain influenza genes previously identified in viruses from finch-like birds. Because wild and domestic songbirds interact with humans and poultry, we investigated the susceptibility and transmissibility of subtype H7N9 in these species. Finches, sparrows, and parakeets supported replication of a human subtype H7N9 isolate, shed high titers through the oropharyngeal route, and showed few disease signs. Virus was shed into water troughs, and several contact animals seroconverted, although they shed little virus. Our study demonstrates that a human isolate can replicate in and be shed by such songbirds and parakeets into their environment. This finding has implications for these birds’ potential as intermediate hosts with the ability to facilitate transmission and dissemination of A(H7N9) virus.

The emergence of novel influenza strains from the avian reservoir remains a constant threat to human and animal health, as was recently illustrated by human infections with novel and wholly avian influenza A(H7N9) viruses in China. These viruses show little virulence in birds but can cause severe illness in humans (*1*,*2*). Of the 134 confirmed human cases reported as of August 2013, >30% have been fatal (*3*,*4*). In the 3 index case-patients, the illness progressed to acute respiratory distress syndrome and death (*1*), and most persons with confirmed infections required hospital care (*2*,*5*). Retrospective epidemiologic analyses showed >75% of affected patients had had contact with domestic poultry (*6*,*7*), a common source of zoonotic transmission of influenza (*8*). Several of the A(H7N9) virus internal genes (polymerase basic protein [PB] 1, matrix, nonstructural protein, and nucleoprotein) originated from the H9N2 subtype commonly found in chickens. When chickens and quail were inoculated with A(H7N9) isolated from humans, they shed the viruses to high titers but had little or no clinical disease (*9*,*10*). Thus, poultry appears to be a reservoir for A(H7N9) viruses and a source of human infections. Yet, multiple lines of evidence suggest avian species other than the usual suspects (waterfowl and poultry) contributed to the emergence of these novel H7N9 viruses: first, H7N9 has been isolated from nonpoultry birds (pigeons) in Chinese live-bird markets (*11*); second, 2 genes (PA, PB2) in an initially characterized human isolate (A/Anhui/1/2013) were most closely related to viruses isolated from bramblings (finch-like birds of the large order Passeriformes) (*12*); and third, the matrix, polymerase acidic protein [PA], PB1 and PB2 gene segments from additional human isolates appear to have been donated by A/brambling/Beijing/16/2012 (H9N2)-like virus(es) (*13*). Therefore, songbirds and other small, terrestrial birds could have been directly involved in the genesis of novel A(H7N9) viruses and subsequent infection in humans.

Songbirds are common household pets and are in close contact with humans and domesticated animals. Their wild counterparts also are likely to interact with poultry in backyard farms and in many farming sectors (*14*,*15*). Consequently, we examined the replication and transmission of the human isolate A/Anhui/1/2013 (H7N9) in wild and domesticated small birds. A/Anhui/1/2013 was isolated from one of the initially reported human case-patients (*1*) and is closely related to many of the avian isolates that have been recovered (*12*). For this study, we chose 3 species of Passeriformes (zebra finches, society finches, and sparrows), which are related to the bramblings described previously. We also studied the parakeet (budgerigar; order Psittaciformes), a bird found in the wild and in households as a pet, that is known to support the replication of other subtypes of influenza (*16*–*18*). The study was conducted during June and July 2013 at St. Jude Children’s Research Hospital (Memphis, TN, USA).

## Methods

### Virus and Facilities

A/Anhui/1/2013 (H7N9), A/Vietnam/1203/04 (H5N1), and A/songbird/Hong Kong/SB102/2001 (H3N8) viruses were propagated and titrated in chicken eggs as described (*15*,*19*,*20*). Pooled allantoic fluid was used for each study. A/Anhui/1/2013 (H7N9) used in these experiments was passaged 3 times in eggs from the original patient sample, and the sequence of the virus inoculum corresponded to Global Initiative on Sharing Avian Influenza Data accession no. EPI_ISL_138739. Experiments were performed under Biosafety Level 3+ containment in accord with the federal regulations (US Department of Agriculture 9 CFR 121 and 7 CFR 331, www.aphis.usda.gov/programs/ag_selectagent/downloads/FinalRule3-18-05.pdf).

### Animals

Commercially acquired zebra finches (*Taeniopygia guttata*), society finches (*Lonchura striata domestica*) and parakeets (*Melopsittacus undulates*) and wild-caught house sparrows (*Passer domesticus*), were quarantined for 1–3 weeks and displayed no signs of disease before the experiment. We serologically tested 3 or 4 sentinel birds of each species (excluding sparrows because of limited availability) for influenza antibodies (H3, H5, H7) by hemagglutination inhibition (HI) assay and found them to be antibody negative. Swabs taken on day 0 were negative for virus isolation in eggs. Food was provided ad libitum, and a minimum of 0.25 L of water was provided daily with a full change of water every 48 h. All birds within a given group shared the same water and food troughs. Groups of birds were inoculated intranasally, intraocularly, and orally with 10^5^ log_10_ 50% egg infectious dose (EID_50_) of pooled allantoic fluid containing A/Anhui/1/2013 (H7N9) in 100 μL of phosphate-buffered saline. The inoculated animals were co-housed with 2 (parakeets) or 3 (finches, sparrows) naïve, direct-contact birds. Each bird’s oropharynx and cloaca were swabbed every second day for 10 days. For each sample, virus was isolated and titrated in eggs in triplicate (3 eggs/sample, 100 μL each of 6 serial log_10_ dilutions) as described (*15*,*20*). All animal experiments were approved by the St. Jude Animal Care and Use Committee and complied with all applicable US regulations.

### Necropsy

At 3 days post inoculation (dpi), 2 finches from each group and 1 sparrow were euthanized for necropsy. Parakeets were excluded from euthanasia and subsequent necropsy because of limited numbers. To prevent cross-contamination, organs were harvested in the following order, and instruments were cleaned after each organ was sampled: brain, eye, lung, trachea, small intestine, and large intestine. Tissues were homogenized, and virus was isolated and titrated in eggs as described (*15*,*20*). Birds that were found dead underwent similar necropsy, but only brain, lung, and combined (small and large) intestinal tissue were collected (*15*,*19*,*20*).

### Serology

At 16 dpi, serum was collected from all surviving animals and tested by HI assay with homologous (A/Anhui/1/2013, H7N9) and heterologous (A/songbird/Hong Kong/SB102/2001, H3N8; A/Vietnam/1203/04, H5N1) viruses by using horse erythrocytes as described (*21*). An HI titer >20 was considered indicative of recent infection with A(H7N9) virus, whereas titers <20 were considered negative.

### Statistical Analysis

Mean infectious titers and serum antibody titers were compared using the 1-tailed Student *t* test in Excel (Microsoft, Redmond, WA) or GraphPad Prism v5 (La Jolla, CA, USA) software.

## Results

### Replication and Pathogenicity of A(H7N9) Virus

All inoculated birds shed virus, but shedding was confined to the oropharynx; no virus was isolated at any time from the cloaca. Shedding was highest in the 2 finch species at 2 dpi, and virus titers shed by these birds were 1.5–1.9 log_10_ higher than those from the sparrows or parakeets (p<0.001). Subsequently, society finches showed higher shedding than sparrows at 4 dpi (p<0.001), but the remaining groups did not differ in levels of virus shedding. Virus was shed for 6 days by finches and parakeets and for 4 days by sparrows, and >80% of the zebra finches and parakeets continued to shed virus at 6 dpi (Table 1). Virus had cleared in all inoculated animals by 8 dpi. One sparrow and 1 zebra finch were found dead at 3 and 6 dpi, respectively (Table 1), but only the sparrow had shown clinical signs of disease (lethargy; loose, discolored feces; ruffled feathers). Surviving inoculated birds were free of disease signs, although a slight decrease in food consumption and emptying of food troughs was observed at 6–9 dpi among zebra finches. In conclusion, all 4 species of small birds tested were susceptible to infection with A/Anhui/1/2013.

**Table 1:** Oropharyngeal and cloacal virus titers in birds inoculated with influenza A(H7N9) virus*

Species	Titer from oropharyngeal swab†				No. deaths‡
	2 dpi	4 dpi	6 dpi	8 dpi	
Zebra finch	4.8 ± 0.5 (7/7)	3.8 ± 1.3 (5/5)	2.9 ± 1.0 (5/5)§	<	1/5
Society finch	4.9 ± 0.5 (7/7)	3.9 ± 0.7 (5/5)	1.0 ± 0.0 (1/5)	<	0/5
Sparrow	3.0 ± 0.5 (6/6)	3.0 ± 0.7 (3/4)	<	<	1/5
Parakeet	3.4 ± 0.5 (5/5)	3.9 ± 1.6 (4/5)	2.6 ± 0.1 (4/5)	<	0/5

*dpi, days post inoculation; <, below the limit of detection (<0.75 EID_50_/mL); EID_50_, 50% egg infectious dose. †Log_10_ EID_50_/mL. Data are the mean ± SD of positive samples (no. birds shedding virus/total no. sampled at the indicated time point). All cloacal samples were below the limit of detection at all time points. ‡Number of animals found dead out of the total for each group; excludes necropsied animals §Includes the animal found dead on this day.

### Shedding of Virus into Water

Each day for 6 days, water was sampled from the communal trough shared by birds within each cage group, and virus was titrated in eggs. Virus was detected in all water troughs and on multiple days (Figure). Both finch species shed virus into the water on every postinoculation day studied, with the exception of 3 dpi in the zebra finches. Virus was not shed into the water until 3 dpi by the sparrows and parakeets. Zebra finches tended to shed more virus into the water than did sparrows or parakeets, and mean titers across of all sampled times differed significantly between these groups (p<0.05). However, the possibility that bird groups consumed different levels of water on any given day could not be normalized.

**Figure:**
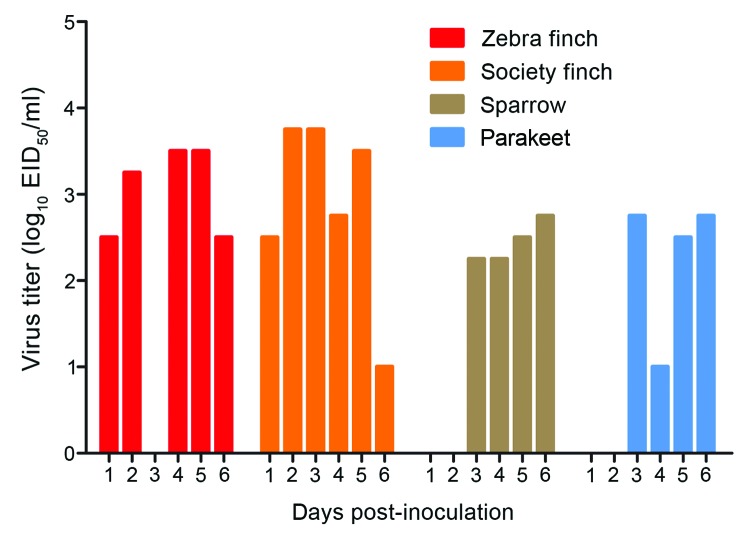
Virus shedding into water trough. A 500-μL sample of water was collected daily for 6 days, and virus was titrated in chicken eggs. The lower limit of detection was 0.75 50% egg infectious dose/mL.

### Shedding by Direct Contacts

In the songbirds and parakeets, A/Anhui/1/13 virus was not highly transmissible to direct-contact animals. A single contact zebra finch showed trace amounts of virus at 2 and 4 dpi, and 2 sparrows showed trace amounts of virus at 4 dpi. Contact parakeets remained virus negative. In contrast, 1 contact society finch shed high titers of virus from 2 dpi (10^5.8^ log_10_ EID_50_/mL) through 8 dpi (Table 2). As with the inoculated animals, direct contacts shed virus only by the oropharyngeal route.

**Table 2:** Shedding of influenza A(H7N9) virus by direct contact among birds*

Species	Titer from oropharyngeal swab†			
	2 dpi	4 dpi	6 dpi	8 dpi
Zebra finch	1.0 ± 0.0 (1/3)‡	1.0 ± 0.0 (1/3) ‡	<	<
Society finch	5.8 ± 0.0 (1/3)	3.5 ± 0.0 (1/3)	2.3 ± 0.0 (1/3)	2.8 ± 0.0 (1/3)
Sparrow	<	1.9 ± 1.2 (2/3)	<	<
Parakeet	<	<	<	<

*dpi, days post inoculation; <, below the limit of detection (<0.75 EID_50_/mL); EID_50_, 50% egg infectious dose. †Log_10_ EID_50_/mL. Data are the mean ± SD of positive samples (no. birds shedding/total no. sampled at the indicated time point). All cloacal samples were below the limit of detection at all time points. ‡Sample contained trace amount of virus: 1 or 2 of 3 inoculated eggs was positive at the lowest serial dilution.

### Isolation of Virus from Organs

Organs from inoculated birds were recovered 3 dpi, and virus was isolated and titered in chicken eggs. The sparrow that underwent necropsy showed trace virus only in the lungs (Table 3). Both finch species showed high virus titers in the trachea (4.5–4.6 log_10_ EID_50_/mL). In the zebra finches, virus was observed only in the tracheas, consistent with swab findings, but 1 of 2 society finches showed trace amounts of virus in the brain and eye, whereas the other had trace amounts in the small and large intestine and high lung virus titer (5.8 log_10_ EID_50_/mL) (Table 3). Two donor birds (one sparrow and one zebra finch) died during the experiment and underwent necropsy. No virus was isolated in any of the sparrow’s organs. However, in the zebra finch, virus was detected in brain, lung, and intestine (2.5, 5.5, and 2.5 log_10_ EID_50_/mL, respectively), suggesting that finches are vulnerable to extrapulmonary A(H7N9) virus infection (Table 3).

**Table 3:** Influenza A(H7N9) virus replication in organs of inoculated birds*

Species	Organ titer†					
	Brain	Eye	Trachea	Lung	Small intestine	Large Intestine
Zebra finch	<	<	4.5 ± 0.0 (2/2)	<	<	<
Society finch	2.5 ± 0.0 (1/2)	1.0 ± 0.0 (1/2)	4.6 ± 2.7 (2/2)	5.8 ± 0.0 (1/2)	1.0 ± 0.0 (1/2)‡	2.5 ± 0.0 (1/2)
Sparrow	<	<	<	1.0 ± 0.0 (1/1)‡	<	<

*<, below the limit of detection (<0.75 EID_50_/mL.) EID_50_, 50% egg infectious dose. †Log_10_ EID_50_/mL. Data are the mean ± SD of positive (>0.75 EID_50_/mL) samples (no. birds shedding/total no. sampled at the indicated time point). ‡Sample contained trace amount of virus: 1 or 2 of 3 inoculated eggs was positive at the lowest serial dilution.

### Rates of Seroconversion

All surviving birds were tested for seroconversion by HI assay with serum collected at 16 dpi (Table 4). Among inoculated birds, 100% of society finches and sparrows seroconverted to homologous virus, as did 75% of zebra finches and 80% of parakeets. Mean HI titers in inoculated birds ranged from 4.8 to 6.9 log_2_ (HI 30–140) (Table 4). All contact zebra finches seroconverted, but only 1 of 3 society finches and 2 of 3 sparrows seroconverted. No seroconversion of contact parakeets was observed. Mean HI titers in contact animals that seroconverted were 4.3–6.3 log_2_ (HI 20–80). Mean titers were highest in society finches and lowest in parakeets, although they did not differ significantly in inoculated versus contact groups or according to species. HI titers to heterologous human subtype H5 and songbird subtype H3 viruses were negative (Table 4).

**Table 4:** Seroconversion of birds to influenza A(H7N9)*

Species, exposure	HI titer†		
	Baseline‡	Homologous virus	Heterologous virus§
Zebra finch			
Inoculated	<	5.3 ± 1.0 (3/4)	<
Contact	<	4.3 ± 0.0 (3/3)	<
Society finch			
Inoculated	<	6.9 ± 0.9 (5/5)	<
Contact	<	6.3 ± 0.0 (1/3)	<
Sparrow			
Inoculated	ND	5.8 ± 0.6 (4/4)	<
Contact	ND	4.3 ± 0.0 (2/3)	<
Parakeet			
Inoculated	<	4.8 ± 0.6 (4/5)	<
Contact	<	< (0/2)	<

*HI, hemagglutination inhibition; <, below the limit of detection (serum dilution<1:20); homologous virus: A/Anhui/1/2013(H7N9); heterologous viruses: A/Songbird/Hong Kong/SB102/2001 (H3N8) and A/Vietnam/1203/04 (H5N1); ND, not determined because of limited number of available birds; HA, hemagglutinin. †Reciprocal value (log_2_/50 μL) of the highest titer that inhibited 4 HA units of virus (no. seropositive animals/total no. sampled). Data are the mean ± SD of positive samples. ‡Baseline HI titers (3 birds/group) were obtained before virus challenge. §HI titers to heterologous viruses were determined in the serum (16 dpi) from the same number of birds used to determine the HI titers to the homologous virus.

## Discussion

We assessed parakeets and 3 species of songbirds for their susceptibility to avian-origin A(H7N9) (A/Anhui/1/2013) virus and found that they were highly susceptible to infection with this isolate. Shedding was limited to the oropharynx, which may have reduced contact transmission; however, at most times sampled, the water troughs contained large amounts of virus. Furthermore, low-pathogenicity influenza viruses (those lacking a multibasic cleavage site in the hemagglutinin protein), such as the A(H7N9) isolate used here, remain stable in water longer than their highly pathogenic counterparts (*22*) and could therefore serve as an inoculum for cage mates. Despite little shedding in the contacts, seroconversion of at least 1 contact animal in each of the finch and sparrow groups indicates exposure with antigenic epitopes of the subtype H7 hemagglutinin. The minimal transmission to direct contacts observed in our study is consistent with previous observations of avian influenza virus infection of songbirds (*15*,*20*,*23*–*25*). The parakeets in particular showed no contact animal shedding or seroconversion, a phenomenon we have previously observed with an A(H3N8) isolate from a songbird (R.G. Webster et al., unpub. data). This observation may have implications for risk assessment of this species in the pet bird trade; however, the lack of proper influenza transmission data with this species in the literature warrants additional studies to confirm the validity of this observation. One direct-contact society finch shed virus equivalent to titers in inoculated birds and shed for a longer period, which suggests that efficient transmission and replication in contact animals, although rare, is possible. The ability of these small birds to harbor and shed A(H7N9) viruses, usually with few signs of illness, creates a substantial potential for transmission to humans, as well as to poultry and wild birds.

Interspecies transmission has not yet been investigated, and the extent to which A(H7N9)-infected finches, sparrows, and parakeets may transmit virus to other species, including through shared water sources, is unknown. Two host groups have the greatest potential interaction with small birds. The first is domesticated poultry, primarily chickens but also a wide variety of gallinaceous and game birds. The peridomestic nature of songbirds facilitates an interaction with poultry in large production facilities and in backyard farms (*14*,*15*). In these cases, they may share common food and water sources (*15*). The interspecies transmission of influenza from songbirds to poultry is not without precedent. Nestorowicz et al. proposed that highly virulent A(H7N7) isolates from starlings and chickens were closely related and indicated that the virus had been transmitted between these 2 species (*23*). Forrest et al. experimentally demonstrated waterborne transmission of highly pathogenic A(H5N1) virus from chickens to starlings (*15*), although the high death rate of the inoculated birds might have limited the degree of interspecies transmission. In the case of the low-virulence A(H7N9) viruses, most inoculated birds shed virus while remaining clinically healthy. The absence of illness and death in A(H7N9)-infected birds has implications for a greater quantity and duration of virus shedding into the environment, as well as higher activity levels and likelihood of interaction with other susceptible hosts. Preliminary data suggest that chickens and quail are highly susceptible to infection with this human A(H7N9) isolate and that the virus is readily transmitted by direct contact (*9*,*10*). The high titers recovered from the shared water troughs of all species tested in our study suggest that the virus could be transmitted to poultry and other birds through this route. Therefore, reduced interaction of domestic poultry with wild passerine birds is advisable, although this precaution might not be feasible in developing countries where numerous backyard farms lack biosecurity. Comprehensive biosecurity also is often lacking at large poultry farms in industrialized and developing countries and should be enhanced to limit the access of wild songbirds to the poultry’s water and food sources.

A second host group with high potential to interact with songbirds and other small terrestrial birds are humans. Finches, sparrows, and parakeets are not only common in the wild but are popular pets worldwide (*26*). They are often sold in the live-bird markets of eastern Asia, where the risk for zoonotic influenza transmission of H7N9 and other influenza subtypes is already established (*26*,*27*). Such pet birds may be procured from the wild and may have been exposed to a variety of pathogens before entering the market chain (*9*). In China, the keeping of pet birds is associated with luck (*28*) and is common among elderly men, who often stroll through the parks with their caged birds (*28*,*29*). In caring for such pets, their owners could become infected by virus contaminated drinking water or from fomites on the feathers (deposited while bathing in water troughs or from saliva while preening) (*30*). This same demographic group (elderly men) experienced disproportionate rates of illness and death from A(H7N9) infection in China (*31*). A recent epidemiologic study by Rivers et al. concluded that the comparatively higher rates of infection among the elderly than among younger age groups cannot be entirely attributed to increased exposure to poultry. Furthermore, they assert that an “as-yet unknown epidemiological or immunological feature” may explain the high infection rates among older persons (*27*), which leaves open the contribution of alternate exposure sources, such as infected pet birds, as a possibility. Virus also could be transmitted to humans through religious ceremonies, such as the Buddhist practice of “merit release,” in which a songbird is purchased, held to the face, kissed, and released (*25*). Simply owning a pet bird increased a household’s rate of seroconversion during the 2003 A(H7N7) outbreak in the Netherlands (*32*). Although completely avoiding contact with pet birds during an avian influenza outbreak might not be feasible, surveillance of such species in the markets, and perhaps in the wild, would help to identify or rule out previously unsuspected hosts that might support or disseminate emerging viruses.

To expand on our findings, future studies should include the examination of genetic changes in the human A(H7N9) virus during replication and transmission in the songbirds and parakeets. The human isolate we used contained genetic markers (HA: S138A, G186V, and Q226L; PB2: E627K) indicating adaptation to mammals (*33*). Sequence analysis of virus shed by the small birds in our study might indicate which species would be most receptive to transmission. The loss of the markers of mammalian adaptation and reversion to an avian-like genotype might predispose the virus to transmission from the small birds to poultry and other birds, rather than to humans. Additionally, studies that assess the sharing of housing, water, and food by inoculated songbirds and poultry (particularly chickens or quail) would shed light on the interspecies transmission potential of A(H7N9) viruses.

Our demonstratration that parakeets and multiple species of songbirds are susceptible to influenza A(H7N9) virus isolated from humans during the recent outbreak in China further supports the possible contribution of songbirds and parakeets to the ecology, maintenance, and transmission of novel A(H7N9) viruses. Finally, they lead us to propose that finches, sparrows, and parakeets may be intermediate hosts and sources of A(H7N9) viruses and that their frequent interaction with wild birds, domestic poultry, and humans renders them a particular risk factor in the emergence and transmission of novel influenza strains.
